# Identification of risk factors for incident cervical insufficiency in nulliparous and parous women: a population-based case-control study

**DOI:** 10.1186/s12916-022-02542-7

**Published:** 2022-10-12

**Authors:** Lili Meng, Sara Öberg, Anna Sandström, Chen Wang, Marie Reilly

**Affiliations:** 1 Department of Gynecology and Obstetrics, the Sun Yat-Sen Memorial Hospital of Sun Yat-Sen University, Guangzhou, 510120 Guangdong China; 2 Department of Medical Epidemiology and Biostatistics, Karolinska Institutet, Nobels vag 12A, 171 77 Stockholm, Sweden; 3 Department of Epidemiology, Harvard T.H. Chan School of Public Health, Boston, USA; 4 Clinical Epidemiology Division, Department of Medicine, Karolinska Institutet, Stockholm, Sweden; 5 Department of Women’s Health, Karolinska University Hospital, Stockholm, Sweden

**Keywords:** Multifetal gestation, Reproductive history, Miscarriage, Preterm birth, Surveillance, Twin pregnancy

## Abstract

**Background:**

Cervical insufficiency is one of the underlying causes of late miscarriage and preterm birth. Although many risk factors have been identified, the relative magnitude of their association with risk in nulliparous versus parous women has not been well demonstrated, especially for incident cervical insufficiency (ICI). The aim of this study was to investigate and compare the magnitude of the association of ICI with predictive factors in nulliparous and parous women, and to further investigate various aspects of obstetric history for parous women.

**Methods:**

Pregnant women with a first diagnosis of cervical insufficiency were compared to a random sample of control pregnancies from women with no diagnosis by using Swedish national health registers. Demographic, reproductive, and pregnancy-specific factors were compared in case and control pregnancies, and relative risks presented as odds ratios (OR), stratified by nulliparous/parous. Independent associations with ICI were estimated from multivariable logistic regression. Associations with obstetric history were further estimated for multiparous women.

**Results:**

A total of 759 nulliparous ICI cases and 1498 parous cases were identified during the study period. Multifetal gestation had a strong positive association with ICI in both groups, but of much larger magnitude for nulliparous women. The number of previous miscarriages was also a much stronger predictor of risk in nulliparous women, especially for multifetal pregnancies. History of preterm delivery (<37 weeks’ gestation) was an independent predictor for parous women, and for those whose most recent delivery was preterm, the association with ICI increased with each additional week of prematurity. A previous delivery with prolonged second stage of labor or delivery of a very large infant were both inversely associated with risk of ICI in the current pregnancy.

**Conclusions:**

The differences in importance of predictive risk factors for incident cervical insufficiency in nulliparous and parous women can help resolve some of the inconsistencies in the literature to date regarding factors that are useful for risk prediction. Stratifying on parity can inform more targeted surveillance of at-risk pregnancies, enable the two groups of women to be better informed of their risks, and eventually inform screening and intervention efforts.

**Supplementary Information:**

The online version contains supplementary material available at 10.1186/s12916-022-02542-7.

## Introduction

Cervical insufficiency refers to a condition in which patients present with a dilated cervix in the mid trimester of pregnancy, potentially with protruding membranes, in the absence of uterine contractions or vaginal bleeding [1]. It is one of the most important causes of late miscarriage and spontaneous preterm birth (sPTB, < 37 weeks’ gestation) [2]. Since women with a history of these conditions are more likely to be examined for cervical length in a subsequent pregnancy, cervical insufficiency is more easily detected in women with previous late miscarriages and in parous women. While “a painless cervical dilation, leading to recurrent second trimester births in the absence of other etiologies in otherwise normal pregnancy” is an accepted definition [3], there is a lack of consensus in published studies regarding both the definition and diagnosis [1, 3, 4] of cervical insufficiency, presenting a challenge for decisions regarding treatment. Cervical cerclage is accepted as an effective intervention for pregnancies where a history of previous pregnancy loss or extreme premature delivery indicates a risk of cervical insufficiency [1]. Such intervention requires women at risk to be identified before the cervix becomes too short or dilated. Several factors that could predict the risk for cervical insufficiency have been identified, with a history of late miscarriage or preterm birth being particularly important, and consequently included in some screening protocols [5]. Other reported predictors include a history of cervical procedures or prolonged second stage of labor [6, 7] and polycystic ovary syndrome (PCOS) [8, 9]. However, studies disagree on the importance of the factors investigated, and the definition and identification of cervical insufficiency remains elusive. Overcoming these difficulties is important in order to resolve the controversies concerning cerclage [10] and to realize the potential of this intervention and progesterone to reduce the risk of pregnancy loss or preterm birth [11].

To date, the independent associations of the various reported predictors of risk for cervical insufficiency have not been investigated in a population-based study, especially for incident cervical insufficiency (ICI), and differences between nulliparous and parous women have rarely been discussed in the literature. The objective of this study was to take advantage of a data linkage of Swedish national population health registers containing demographic information, reproductive history and surgical procedures, to identify independent correlates of ICI in nulliparous and parous women.

## Methods

### Data sources

This population-based case-control study was based on a linkage of the Swedish Medical Birth Register (MBR) to the National Patient Register (NPR), made possible by the unique personal identification number assigned to all Swedish citizens and residents. The MBR has recorded almost every delivery in Sweden since 1973, with prospectively collected information on maternal characteristics, medical and reproductive history, and information from antenatal, delivery, and postnatal care, including diagnoses and procedures coded according to the Swedish version of the International Classification of Diseases (ICD). The NPR, which also uses ICD codes, has nation-wide inpatient coverage from 1987, with specialist outpatient visits included from 2001. Pregnancies of women with fertility problems (more than one year attempting to conceive) or by assisted reproduction were excluded as these have higher rates of many obstetric complications [12, 13], and perhaps interventions, and are the focus of a separate investigation.

### Study design

The study considered case and control deliveries in the MBR from 1992 to 2012 to allow for at least 5 years for ascertainment of prior hospitalizations and procedures in the NPR. Delivery information, pregnancy details, and reproductive history were extracted from the MBR for all women with known date of birth and whose delivery records were consistently coded with respect to dates and singleton/multiple gestation. In addition, maternal complications during pregnancy were identified from ICD-9 and ICD-10 diagnosis or procedure codes recorded in the NPR during the pregnancy, which was defined as the interval from the last menstrual period date to the delivery date. The different diagnosis and procedure systems in use over the time span of the registers are shown in Additional file 1: Table S1. The first record of a diagnosis code for cervical insufficiency, or a procedure code for cervical cerclage, was defined as incident cervical insufficiency (ICI) and used to extract the cases for this study. A random sample of control deliveries was selected from pregnancies without cervical insufficiency, using a case control ratio of 1:10. The delivery identified as the case or control record will be referred to as the “index delivery”, to distinguish it from prior deliveries of the same woman. In addition to the reproductive and medical history information collected during the index pregnancy, records from previous pregnancies were also identified.

### Definition of factors investigated

#### Demographic/maternal characteristics

For each pregnancy, information was available on mother’s country of birth, smoking status, weight and height at the first antenatal visit, and age at delivery. Body mass index (BMI) was calculated from weight and height and classified into 3 groups (<25kg/m^2^, 25–30kg/m^2^, and ≥30kg/m^2^). Mother’s country of birth was grouped into three categories based on similar crude associations with ICI: Sweden, Africa and Eastern Mediterranean, and other regions. Smoking status, which was ascertained early in the pregnancy and at gestational week 32, was classified as any/no report at either of these time points. Other characteristics considered were pre-gestational diabetes, chronic hypertension, and polycystic ovary syndrome (PCOS).

#### Pregnancy-specific factors

Risk factors specific to the index pregnancy were parity (classified into 3 groups: 0, 1, and ≥2) and multi-fetal gestation.

#### Cervical procedures and reproductive history

Information on a woman’s history of cervical procedures was obtained by examining the NPR data prior to the index pregnancy for records of hysteroscopy, excision/destruction of a lesion of the cervix, and dilation and curettage or cervical excision. Women were also classified as having a history of “any cervical procedures.” The number of previous miscarriages, which were classified into four categories: 0, 1, 2, and ≥3, was obtained from the woman’s self-report at enrolment in antenatal care. Information about previous pregnancies leading to deliveries was obtained from a woman’s previous records in the MBR and included preterm deliveries, preterm premature rupture of membranes (<37 weeks’ gestation; PPROM), prolonged second stage of labor (ICD codes: >3h for nulliparous and 2h for parous), macrosomia (birthweight > 4000g), and gestational diabetes, each coded as yes/no. In addition to any history of preterm delivery, the number of previous preterm deliveries was also considered, and for the delivery prior to the index, we created a variable “weeks-to-term” to capture the seriousness of prematurity, by subtracting the gestational week at premature delivery from 37.

### Statistical analysis

We first compared the distributions of characteristics between cases and controls, stratified by nulliparous or parous, using percentages for categorical variables and means and standard deviations for continuous variables. Categorical variables were defined as outlined above, and maternal age, mother’s height and BMI, and “weeks to term” were continuous variables. The crude association of each variable with ICI was presented as an odds ratio (OR) with 95% confidence interval from a logistic regression model, where the OR is relative to the reference level for categorical variables and per unit change for continuous variables. The “weeks to term” was set to zero for term deliveries (gestational week ≥37) so that these served as the reference group. To assess the independent associations, predictors with a statistically significant crude association with ICI were included in multivariable logistic regression models, together with year of delivery to adjust for potential changes over calendar time. The model for parous women included reproductive history variables in addition to the variables in the model for nulliparous women. Pregnancies missing any of the considered predictors were excluded from the multivariable analysis. Interaction terms were used to investigate whether the associations differed for singleton and multifetal pregnancy. All data preparation and analyses were conducted in SAS software version 9.4 (SAS institute Inc., Cary, NC, USA).

## Results

### Cases identified

During the study period (1992–2012), there were 2257 cases of incident cervical insufficiency (ICI) and 24,628 controls available for analysis. Of the 2257 cases, 2206 were identified by ICD diagnosis code only, 27 were identified by procedure code only, and 24 had both diagnosis and procedure codes. Approximately one-third of the cases (759) occurred in nulliparous women and two-thirds (1498) in parous women.

### Description and crude associations

Table 1 compares the characteristics of cases and controls, stratified by parity, with crude odds ratios for the associations with ICI. There were notable differences between nulliparous and parous women, with the former showing more pronounced associations of ICI with multi-fetal gestation and a history of miscarriage. Crude ORs for demographic factors were similar in nulliparous and parous women, except for smoking during pregnancy, which was only statistically significant in the parous group. Cervical procedures were associated with an approximate doubling of the risk in both groups (nulliparous OR: 2.48, 95%CI: 1.80–3.42; parous OR: 1.82, 95%CI: 1.38–2.38). For parous women, characteristics of the previous delivery with the largest positive associations were PPROM and preterm birth, and there were negative associations with previous experience of prolonged second stage of labor and macrosomia.

**Table 1:** Descriptive statistics of incident cervical insufficiency (ICI) cases and controls, stratified by parity

	Nulliparous (***N***=11,087)			Parous (***N***=15,798)		
	CI (***n***=759) (%)	Control (***n***=10,328) (%)	Crude OR (95% CI)	CI (***n***=1498) (%)	Control (***n***=14,300) (%)	Crude OR^a^ (95% CI)
**Height (cm)**	165.4 ± 6.88	166.5 ± 6.36	0.97 (0.96, 0.99)	165.2 ± 6.39	166.2 ± 6.27	0.98 (0.97, 0.99)
Missing, *N* (%)	107 (14.10)	813 (7.87)	~	146 (9.75)	1065 (7.45)	~
**BMI**	24.68 ± 4.95	23.85 ± 4.26	1.05 (1.04, 1.07)	24.75 ± 4.80	24.56 ± 4.38	1.01 (1.00, 1.02)
Missing, *N* (%)	148 (19.50)	1412 (13.67)	~	218 (14.55)	1900 (13.29)	~
**Age (years)**	29.02 ± 5.08	27.38 ± 5.88	1.07 (1.06, 1.09)	32.02 ± 5.06	30.96 ± 4.78	1.05 (1.04, 1.07)
**Mother’s country of birth:**						
Sweden (reference)	540 (71.15)	8478 (82.09)		1020 (68.09)	11335 (79.27)	
African and Eastern Mediterranean	97 (12.78)	589 (5.70)	2.79 (2.26, 3.44)	171 (11.42)	1116 (7.80)	1.7 (1.45, 2.00)
Other	107 (14.10)	1168 (11.31)	1.7 (1.41, 2.04)	295 (19.69)	1782 (12.46)	1.83 (1.60, 2.08)
**Previous miscarriage number:**						
**None**	397 (52.31)	9068 (87.80)		795 (53.07)	10910 (76.29)	
1	177 (23.32)	1081 (10.47)	3.46 (2.92, 4.09)	367 (24.50)	2496 (17.45)	2.05 (1.81, 2.32)
2	126 (16.60)	140 (1.36)	16.16 (12.91, 20.22)	182 (12.15)	659 (4.61)	3.66 (3.09, 4.32)
≥3	59 (7.77)	39 (0.38)	27.31 (19.83, 37.60)	154 (10.28)	235 (1.64)	9.63 (7.90, 11.74)
**Any miscarriage**	362 (47.69)	1260 (12.20)	6.21 (5.42, 7.12)	703 (46.93)	3390 (23.71)	2.92 (2.64, 3.23)
**Multifetal gestation**	37 (4.87)	85 (0.82)	6.32 (4.74, 8.44)	57 (3.81)	211 (1.48)	3.05 (2.35, 3.95)
**Smoking during pregnancy**	99 (13.04)	1254 (12.14)	1.12 (0.91, 1.37)	222 (14.82)	1752 (12.06)	1.34 (1.15, 1.55)
Missing	248 (32.67)	412 (27.39)	~	458 (30.57)	4050 (28.32)	~
**Chronic diabetes**	9 (1.19)	51 (0.49)	2.68 (1.46, 4.91)	21 (1.40)	93 (0.65)	2.09 (1.32, 3.30)
**Polycystic ovarian syndrome**	14 (1.84)	100 (0.97)	3.38 (2.31, 4.94)	20 (1.34)	66 (0.46)	2.89 (1.92, 4.34)
**History of dilation and curettage**	18 (2.37)	138 (1.34)	1.89 (1.22, 2.93)	44 (2.94)	162 (1.13)	2.36 (1.70, 3.26)
**History of cervical excision**	47 (6.19)	303 (2.93)	2.22 (1.67, 2.95)	103 (6.88)	473 (3.31)	2.25 (1.82, 2.77)
**History of any cervical procedures**	67 (8.83)	487 (4.72)	1.97 (1.55, 2.50)	142 (9.48)	736 (5.15)	1.96 (1.64, 2.34)
**Parity more than two**	~	~	~	668 (44.59)	5112 (33.75)	1.38 (1.24, 1.53)
**Previous gestational diabetes**	~	~	~	20 (1.34)	167 (1.17)	1.64 (1.00, 2.68)
**Previous delivery with prolonged second stage of labor:**						
**None**				1141 (76.17)	10,315 (72.13)	
1	~	~	~	295 (19.69)	3215 (22.48)	0.83 (0.73, 0.95)
2 or more	~	~	~	62 (4.14)	770 (5.38)	0.73 (0.60, 0.88)
**Previous PPROM**	~	~	~	87 (5.81)	295 (2.06)	2.93 (2.29, 3.74)
**Previous Preterm birth**	~	~	~	301 (20.09)	1216 (8.50)	2.71 (2.35, 3.11)
**Total number of previous preterm births:**						
**None**				1197 (79.91)	13,084 (91.50)	
1	~	~	~	248 (16.56)	1179 (8.24)	2.3 (1.98, 2.67)
2 or more	~	~	~	53 (3.54)	37 (0.26)	15.66 (10.2, 23.9)
**Weeks to term of if premature delivery prior to index**	~	~	~	5.29 ± 4.37	2.91 ± 2.69	1.53 (1.48, 1.58)
**Number of previous cesarean:**						
**None**				1218 (81.31)	11,892 (83.16)	
1	~	~	~	262 (17.49)	2289 (16.01)	1.12 (0.97, 1.29)
2 or more	~	~	~	18 (1.20)	119 (0.83)	1.48 (0.90, 2.44)
**Previous macrosomia**	~	~	~	329 (21.96)	4019 (28.10)	0.72 (0.63, 0.82)

Numbers (and %) for categorical variables, mean (± SD) for continuous variable, number (%) missing where more than 10% in either group, and crude odds ratios

^a^For continuous variables, the odds ratio is for unit change; for all other variables, the reference category is “none”, except for country of birth where Sweden is the reference

### Adjusted associations

All variables with statistically significant crude associations with ICI were investigated for their independent association in multivariable logistic regression models, except for pre-gestational diabetes and PCOS, for which there were insufficient observations for a multivariable analysis. Many of the adjusted associations (Table 2) were similar to the crude values. Multi-fetal gestation was an exception, with the crude association with ICI being substantially diminished after adjustment in parous women (adjusted OR 1.19, 95%C.I. 0.73–1.95), but the adjusted association (OR 8.44, 95% C.I. 5.20–13.68) was even more pronounced than the crude value in nulliparous women.

**Table 2:** Adjusted odds ratios from multivariable logistic regression analysis, stratified by parity

	Nulliparous (***N***=11,087)		Parous (***N***=15,798)	
	Adjusted OR	95% CI	Adjusted OR	95% CI
**Height (cm)**	0.98	0.97, 0.99	0.99	0.98, 1.00
**BMI (kg/m**^**2**^**)**	1.02	0.98, 1.04	0.99	0.97, 1.00
**Age (years)**	1.03	1.01, 1.05	1.01	1.00, 1.03
**Mother’s country of birth (reference=Sweden)**				
Africa or Eastern Mediterranean	2.07	1.53, 2.82	1.55	1.22, 1.98
Other	1.31	1.00, 1.70	1.83	1.51, 2.22
**Previous miscarriages**				
1	4.23	3.43, 5.22	2.03	1.71, 2.40
2	20.57	15.32, 27.63	3.38	2.67, 4.29
3 or more	34.57	21.60, 55.31	6.67	4.93, 9.02
**Multi-fetal gestation**	8.44	5.20, 13.68	1.19	0.73, 1.95
**History of any cervical procedures**	2.48	1.80, 3.42	1.82	1.38, 2.38
**Parity more than two**	~	~	1.23	1.04, 1.44
**Smoking during pregnancy**	~	~	1 .40	1.15, 1.71
**Previous PPROM**	~	~	1.53	1.04, 2.26
**Previous preterm delivery**	~	~	2.06	1.63, 2.61
**Weeks to term of birth prior to index**	~	~	1.47	1.41, 1.54
**Number of previous cesarean sections**				
1	~	~	0.93	0.76, 1.13
2 or more	~	~	0.65	0.30, 1.43
**Number of previous deliveries with prolonged second stage of labor**				
1	~	~	0.75	0.62, 0.92
2 or more	~	~	0.63	0.41, 0.96
**Previous macrosomia**	~	~	0.86	0.72, 1.02

In addition to adjustment for all the variables in the table, all ORs have been adjusted for calendar time. For continuous variables, the odds ratio is for unit change; for all other variables, the reference category is “none”, except for country of birth where Sweden is the reference

The number of previous miscarriages was independently associated with risk of ICI, with evidence of a trend in both nulliparous and parous women, but the adjusted ORs were more pronounced in nulliparous women. An interaction analysis revealed a moderating influence of multiple gestation in nulliparous women: compared to singleton pregnancies with no previous known miscarriage, a history of 1 or more than 1 miscarriage was associated with a 4-fold and 24-fold elevated risk of ICI among singleton pregnancies and a 12-fold and 67-fold elevated risk in multifetal pregnancies. For parous women, in which adjustment was made for factors related to previous deliveries, the associations between history of miscarriage and CI were similar in singleton and multifetal pregnancies.

For parous women, the adjusted OR for a history of preterm birth and for the number of weeks premature of the most recent delivery (“weeks to term”) were similar to the crude ORs. The dose-response effect of the weeks to term was consistent with an analysis that categorized this variable with cut-offs before the 28th, 32nd, and 34th week gestation, with adjusted ORs (95% confidence intervals) of 35.97 (19.91, 64.97), 10.96 (7.83, 15.35), and 6.97 (5.58, 8.71), respectively. The positive association with previous PPROM was diminished in the adjusted analysis, although still statistically significant, while the negative association with previous prolonged second stage of labor was somewhat stronger.

## Discussion

The comparison of the incident cases of cervical insufficiency to the random sample of unaffected pregnancies revealed some important differences in the presence and/or magnitude of associations with predictors in nulliparous and parous women. Increasing maternal age and shorter stature were associated with a reduced risk of ICI in both nulliparous and parous women, with ORs of 1.16 (1.05–1.28) and 1.05 (1.00–1.06) for each additional 5 years of age and 0.86 (0.90–0.95) and 0.95 (0.90–1.00) for a 5-cm difference in height. Other factors found to be independent positive correlates of risk in both nulliparous and parous women were region of birth, previous cervical procedures, and a history of miscarriage. The magnitude of the association with cervical procedures was similar in the two groups, whereas previous miscarriage had a much stronger association for nulliparous women, which was especially noted in multi-fetal gestations. In contrast, for parous women, where further adjustment was made for factors related to previous deliveries, the association of ICI with multi-fetal gestation was much weaker and the independent association with history of previous miscarriages was similar in singleton and multifetal pregnancies. For parous women, a prolonged second stage of labor in a previous delivery and previous delivery of a very large infant were also both inversely associated with risk of ICI in the current pregnancy.

We hypothesized that the negative association of ICI with taller stature may be related to a longer cervix. Weak evidence of an association between maternal height and cervical length was reported from a small study of 146 asymptomatic Turkish women [14], but a larger screening study of 5092 pregnant women in the USA found no such association [15]. BMI, which has previously been reported as a risk factor for sPTB [16, 17], has a weak independent association with ICI only for parous women in our study. There is no clear evidence in the literature regarding the importance of maternal age for the risk of cervical insufficiency. A weak negative association has been reported for older women [18], but the estimate was from a model that assumed common ORs for all other factors for nulliparous and parous women, whereas our study provides strong evidence against this assumption as we found many differences in the presence or magnitude of the risk factors for ICI in the two groups.

Multi-fetal pregnancies are known to be at higher risk of spontaneous preterm birth, and there have been reports of a lack of response to treatment, compared to singleton pregnancies [19, 20]. Recent guidelines [5] report that available data still indicate a risk of preterm birth following cerclage for short cervix (< 25mm) in twin pregnancies, but limited data providing evidence of an advantage for cervix < 15mm. Evidence from a recent clinical trial [18] supports the use of cerclage in twin pregnancies with a dilated cervix. However, further studies of multi-fetal pregnancies are required, as there is a lack of consensus regarding screening protocols or the risks and benefits of cerclage [21–23] and it is notable that current RCOG guidelines [24] present key recommendations only for singleton pregnancies. This could be partly due to differences in parity as we found multi-fetal pregnancy to be a strong predictor of risk for nulliparous but not parous women. This might be explained by the myometrium and cervical tissues having been expanded and thus being more adaptable in a subsequent pregnancy. Evidence that would support this hypothesis comes from a meta-analysis of recurrent preterm birth [25] that reported a 10% rate of preterm deliveries for singleton pregnancies following a preterm twin delivery and only 1.3% following a term twin delivery. Another important difference we found with respect to parity concerns the importance of miscarriage history in singleton and multi-fetal gestation: in nulliparous women a history of 2 or more miscarriages was associated with a 24-fold higher risk of ICI in singleton pregnancies and 67-fold higher risk in multifetal pregnancies, whereas for parous women, the elevated risk for miscarriage history was similar in singleton and multifetal pregnancies. Although the parous women cannot be compared directly with the nulliparous women, as they provide more information (i.e., from their previous deliveries) and use a different model, the findings have value in identifying the important, and different, contribution of these risk factors for ICI in the two groups.

For parous women, we found several aspects of obstetric history to be strongly associated with ICI: a history of sPTB or PPROM were risk factors, in contrast to prolonged second stage of labor that showed an inverse association. As previously noted, a history of sPTB is widely recognized as a risk factor [5] and a small study reported a much higher rate of cervical insufficiency in the subsequent pregnancy of 102 women with PPROM than in 316 without PPROM: 14.7% vs. 1.0%; adjusted OR 3.8, 95% CI 1.2–11.6 [26]. The dose-response that we observed for the “weeks to term” of the delivery prior to the index is consistent with guidelines that recognize early premature delivery in risk evaluation protocols and consider cerclage for women with a history of extreme premature delivery [5]. Although previous prolonged second stage of labor was reported as a risk factor as early as 2006 [6], there is no consistent conclusion regarding the magnitude of the association. A prolonged second stage of labor has previously been investigated for its association with sPTB in the subsequent pregnancy, but these studies either found no association, or concluded that the risk is mediated by cesarean section following prolonged second stage of labor [7, 27, 28]. Prolonged second stage of labor is more commonly diagnosed in women with term birth and thus may rather be a sign of a strong cervix. In our study, a history of prolonged second stage of labor was an independent predictor of a lower risk of ICI. Although our data were insufficient for investigating cesarean section after prolonged second stage of labor (only 16 such cases), it is nonetheless interesting that we did not find any association between previous cesarean section and ICI.

A major strength of our study is that the large population-based data enabled the investigation of ICI in an un-selected national cohort of nulliparous and parous women separately, although the sample sizes were insufficient for further exploration of subgroups with specific reproductive or medical history. A further strength of the study is that pregnancy information was prospectively obtained at attendance for antenatal care and hospitalization information from the NPR, eliminating the potential for recall bias. Further, due to the rich data, we were able to study a broad spectrum of potential predictors for both nulliparous and parous women. Another strength of the study is that we focused on incident cervical insufficiency, while much of the literature is unclear regarding whether incident and/or recurrent cases are being considered [5].

Limitations of our study include the potential for misclassification of the cervical insufficiency diagnosis in both nulliparous and parous women, due to the partly unclear definition of the diagnosis and the inherent difficulty of discriminating between cervical insufficiency and late miscarriage, PPROM, or sPTB, which could occur in a continuum. Given the unclear definition of the diagnosis, the pregnancies captured by explicit ICD codes in the registers may nevertheless be more representative of true cases of cervical insufficiency, and the risk factors identified thus more specific. The diagnostic criteria commonly used are (i) cervical length less than 25 mm according to transvaginal ultrasound measurement before 24 gestational weeks, in a pregnant woman with no uterine contractions and at least one previous preterm birth or late miscarriage, or (ii) cervical length of 20mm or less in a woman with no history of preterm birth or late miscarriage. Since Sweden does not offer universal cervical length screening, the associations with previous miscarriages and previous sPTB in parous women may reflect diagnostic practice and/or detection bias from additional surveillance due to the already known correlation with cervical insufficiency [29]. However, in addition to considering history and number of sPTB, we found a positive association with the number of weeks’ preterm of the most recent delivery, and such dose-response seems unlikely to be the sole result of diagnostic practice or surveillance. These findings suggest that the full history of preterm deliveries prior to various cut-off times for gestational age and their potential interactions with multifetal gestation (such as we observed for miscarriage history) may provide information of value for interventions. Finally, while there was some evidence of changes in the rate of cervical insufficiency diagnosis over time, adjustment for calendar year had no influence on the associations of the predictors.

## Conclusions

The differences we observed for nulliparous and parous women can have important consequences for screening and intervention for cervical insufficiency. The much stronger association with multi-fetal gestation in nulliparous women may contribute to the lack of consensus regarding screening protocols and cerclage for multi-fetal gestation [21, 22]. In addition, the difference in the importance of miscarriage history in singleton and multi-fetal gestation for nulliparous women but not in parous women highlights readily available information that could be used to inform screening protocols. Our work identified aspects of obstetric history that had strong (independent) associations with ICI: inclusion of these in studies of independent risk factors for parous women could help resolve some of the inconsistencies in the literature to date regarding the factors that are important for risk stratification. The identification of different risk factors of importance for nulliparous and parous women has clinical and scientific implications: these two groups of women can be better informed of their risks, and with additional scientific support, screened differently. Finally, stratification by parity in the reporting of risk factors for incident cervical insufficiency would facilitate meta-analyses that can produce more timely synthesized evidence for screening and eventually inform intervention efforts in both groups of mothers.

## Supplementary Information

**Additional file 1: Table S1**. ICD codes used to define medical diagnoses and surgical procedures.

## Data Availability

The datasets generated and analyzed for the purpose of this study are not publicly available due to protocols for the protection of confidentiality of patient data. The data can only be shared in the context of an agreed collaboration and subject to a data-sharing agreement to ensure security of individual-level personal data.

## Abbreviations

BMI: Body mass index
ICI: Incident cervical insufficiency
ICD: International Classification of Diseases
MBR: Medical Birth Register
NPR: National Patient Register
OR: Odds ratio
PPROM: Preterm premature rupture of membranes
PCOS: Polycystic ovary syndrome
sPTB: Spontaneous preterm birth
