# Supplementary material for: Identification of risk factors for incident cervical insufficiency in nulliparous and parous women: a population-based case-control study
Source: BMC Med. 2022 Oct 12;20:348. doi: 10.1186/s12916-022-02542-7 (PMC9555073; doi:10.1186/s12916-022-02542-7)
Supplement: Supplementary file 1 — Additional file 1: Table S1. ICD codes used to define medical diagnoses and surgical procedures. [file 12916_2022_2542_MOESM1_ESM.docx]

**Table S1:** *ICD codes used to define medical diagnoses and surgical procedures*

|  | **International Classification of Diseases** | | |
| --- | --- | --- | --- |
|  | **ICD 10(1997-current)** | **ICD 9(1987-1997)** | **ICD 8(-1986)** |
| **Cervical insufficiency** | O343,P010,N883 | 654F,761A,622F | 63494, 76900 |
| **Chronic diabetes** | E10,E11,E12,E13E14, O24.0,O24.1,O24.2, O24.3,P70.1 | 250 |  |
| **Gestational diabetes** | O24.4,O24.9 | 648A |  |
| **Premature rupture of membrane** | O42.0,O42.1, O42.2,O42.9 | 658B,761B, | 634.95，661.0 |
| **Prolonged second stage of labor** | O63.1 | 662C | 650 |
| **Polycystic ovary syndrome** | E282 | 2564 | 25690 |
|  | **Nordic Classification of Surgical Procedures** | | |
| **Cervical procedure** | 7605(≤1996), MAB00(>1996) | |  |
| **Any hysteroscopy procedure** | ULC，LCB25，LCB28，LCB32，LCG02，LCH20 | | |
| **Excision/destruction of lesion of cervix** | 7380,7381,7312,7313,7320,7321,LDB | | |
| **Dilation & curettage** | LCA06,LCA10,LCA13,LCA16,LCA20,LCA22,LCA30,LCA96,LCA98,LDA00,LDA10, LCB20,LCH00,LCH03,LCH13,LDA20,LDA96 7270 7275,7276,7330 | | |
| **Cervical excision** | 7300,7310,7311,LDC,LDW |  |  |
